# Massive Ascites Causing Presumed Abdominal Compartment Syndrome During Open Heart Surgery With Cardiopulmonary Bypass

**DOI:** 10.7759/cureus.26515

**Published:** 2022-07-02

**Authors:** Michelle Y Chen, Kathleen G Parr

**Affiliations:** 1 Anesthesia and Critical Care Medicine, George Washington University School of Medicine and Health Sciences, Washington, USA

**Keywords:** cardiopulmonary bypass., massive ascites, abdominal hypertension, abdominal compartment syndrome, adult cardiac surgery

## Abstract

Abdominal compartment syndrome (ACS) is a potentially fatal condition and a known cause of morbidity and mortality in critically ill patients. It can be primary, due to abdominal trauma and/or surgical procedures, or secondary, due to excessive abdominal fluid and/or bowel edema. Intra-abdominal hypertension (IAH) is defined as intra-abdominal pressure (IAP) greater than 12 mm Hg. ACS occurs when increased IAP results in organ dysfunction.^ ^Although IAPs are known to increase in cardiac surgery, ACS is uncommon, and reports are limited in the literature. We describe a fatal case of presumed ACS during an aortic valve and root replacement with coronary artery bypass grafting (CABG).

## Introduction

Intra-abdominal hypertension (IAH) and abdominal compartment syndrome (ACS) are known causes of morbidity and mortality in critically ill patients. IAH is defined as intra-abdominal pressure (IAP) greater than 12 mm Hg [1]. ACS occurs when increased IAP results in organ dysfunction [2].

Although increases in IAP occur more commonly in the post-operative period, increases in IAP are known to occur during cardiopulmonary bypass (CPB) and have been implicated in the development of renal failure and mesenteric vascular dysfunction [1,3-8]. The literature on catastrophic IAH and ACS in cardiac surgery, however, is limited to case reports and small studies [9-13]. In one study, the incidence of ACS in cardiac surgery was determined to be about 1% [13]. The definitive cause(s) of ACS and catastrophic IAH is not known and remain subject to investigation [2,7,8,10-12].

Dalfino et al. attempted to identify the risk factors for secondary IAH in cardiac surgery patients. They studied a series of 69 patients, 22 (31.8%) of which developed IAH, and found it to be strongly linked with a higher baseline IAP, high central venous pressure (CVP), positive fluid balance, acute kidney injury (AKI), use of CPB, increased age, and sequential organ failure assessment (SOFA) score [1]. Independent risk factors were the baseline IAP, CVP, and positive fluid balance [1]. The use of CPB and vasoactive drugs were also associated with IAH [1]. Additionally, poor venous drainage has been known to cause increased inferior vena cava (IVC) pressure and ascites.

CPB produces a generalized inflammatory response, and the degree of this response varies from patient to patient [14]. Holmes et al. examined levels of inflammatory mediators in patients who underwent surgery requiring CPB. In one study, those patients with an exaggerated inflammatory response experienced more bleeding, had a larger capillary leak measured via weight gain, and required more respiratory support [15]. In another study in piglet models, hypothermic CPB was found to result in increased fluid third-spacing compared to normothermic CPB [16]. Studies in both humans and piglets have demonstrated that, in contrast to other inflammatory response syndromes, leakage of protein from the vascular space during CPB is low, suggesting another mechanism for extra-vascular fluid accumulation [17,18]. A higher degree of hemodilution during coronary artery bypass grafting (CABG) with normo-volemic hemodilution has resulted in increased IAP [19]. In another study, patients undergoing CPB had higher IAPs and lower abdominal perfusion and coronary perfusion pressures [20]. Given the above-known risk factors for and potential causes of ACS and IAH, we present a case of presumed ACS and catastrophic IAH, followed by a discussion of potential risk factors and causes.

## Case presentation

Our patient was a 69-year-old female (BMI 25.96) with aortic stenosis, heavily calcified aortic root, and coronary artery disease (CAD) of the right coronary artery (RCA) with 80% ostial stenosis and left anterior descending (LAD) coronary artery with 30% proximal narrowing. She also had a history of hypertension (HTN), hyperlipidemia, non-insulin-dependent diabetes (NIDDM), mild anemia (Hg 12.9), and hypothyroidism. She presented for aortic valve replacement (AVR), possible aortic root replacement with coronary reimplantation (Bentall procedure), and possible CABG. Her preoperative workup included a transthoracic echocardiogram (TTE) showing normal right ventricular (RV) function, left ventricle ejection fraction (LVEF) 60-65%, mild concentric left ventricular (LV) hypertrophy, moderate-severe aortic valve stenosis (aortic valve area 1.33 cm2 and mean gradient 32 mm Hg), and mild mitral regurgitation. Her CT angiography of the chest showed calcification of the aortic root. She was considered for trans-arterial AVR; however, this was excluded due to the degree of aortic root calcification seen on CT. Pre-operative labs showed did not show evidence of significant pre-operative renal or hepatic dysfunction or significant anemia (Table 1).

**Table 1:** Preoperative laboratory values

Test	Results	Test	Results
Sodium mmol/L	140	Glucose mg/dL	95
Potassium mml/L	4.3	Calcium mg/dL	9.8
Chloride mml/L	104	Magnesium mEq/L	1.7
Carbon Dioxide mmol/L	29	Creatinine mEq/L	0.9
White Blood Cell Count 10e3/mcL	5.09	Blood Urea Nitrogen mg/dL	19
Hemoglobin gm/dL	12.6	PT seconds	13.8
Hematocrit percent	39.2	PTT seconds	3.2
Platelet Count 10e3/mcL	192	INR	1.07
Urine Bilirubin mg/dL	Negative	Urine Urobilinogen mg/dL	Normal

The patient was taken to the operating room and underwent successful induction of general endotracheal anesthesia. An arterial line was placed in the right radial artery, an 8F introducer with a single lumen introducer catheter was placed in the right internal jugular vein (baseline CVP of 12), and a standard temperature monitoring bladder catheter (with no IAP monitoring) and a transesophageal echocardiogram (TEE) probe were inserted. The pre-incision TEE study was consistent with the pre-operative TTE. The heart was cannulated with an arterial cannula in the ascending aorta and a three-stage venous cannula (per surgeon preference) in the right atrium via the right atrial appendage. There were no issues with venous cannula placement, and it was not visualized on TEE. In addition, a retrograde cardioplegia cannula and a left ventricular vent line were placed. Once CPB was initiated, the aorta was cross clamped and the heart arrested, and the aorta was opened and found to be heavily calcified. There was extensive calcification of the Sinuses of Valsalva and almost total occlusion of the RCA ostium. In addition, there was significant plaque palpated by the surgeon in the left coronary artery (LCA) and left circumflex artery (LCxA). ABG values of interest are listed in Table 2. As it is not standard of care at our institution and immediately available in the OR, lactate levels were not drawn.

**Table 2:** ABG Laboratory Values

ABG Value	After Initiation of Initial CPB Run	Prior to Separation From Initial CPB Run
pH	7.58	7.40
pCO_2_ mmHg	31	41.3
pO2 mmHg	286	658
Base Excess	7	1
Hemoglobin gm/dL	7.1	6.5

Due to the heavy aortic calcifications and coronary disease, the patient underwent a Bentall procedure. A 23 mm bioprosthetic valve was placed into a 26 mm aortic graft and implanted into the aortic annulus after an annular enlargement. The RCA was bypassed with a saphenous vein graft (SVG), and the proximal RCA was tied off. An LCA button was anastomosed to the aortic graft. After cooling to 32℃, rewarming occurred, and the vein graft was de-aired prior to cross-clamp removal. After removal of the cross-clamp, the EKG waveform indicated the presence of coronary and myocardial ischemia, perhaps due to air. The lateral wall of the LV appeared hypokinetic on the TEE exam. Despite 45 minutes of reperfusion time and aggressive de-airing maneuvers, the EKG did not improve, and the LV lateral wall remained hypokinetic. To improve LV function, the surgeon grafted an SVG to the LAD and an obtuse marginal coronary artery (OM), resulting in improved function. The total cross-clamp time was 164 minutes. 5,000 cc of crystalloid, four units of packed red blood cells (PRBC), and 1,000 cc of autologous blood salvage (CS) were given on CPB.

**Figure 1:**
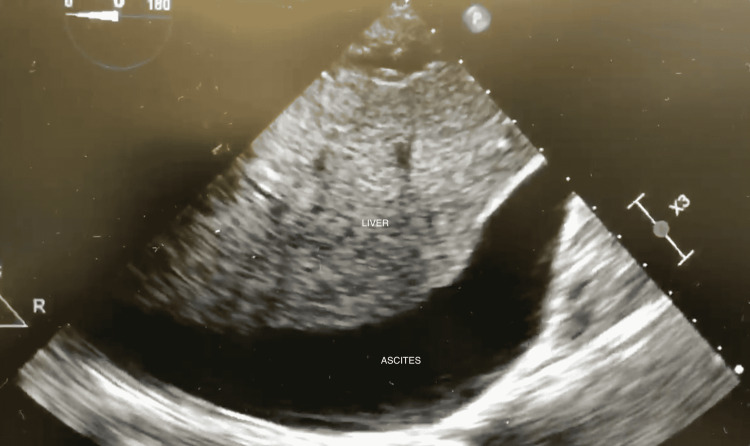
Liver with massive ascites on TEE

To facilitate separation from CPB, the patient was placed on 4 mcg/min of epinephrine. She was initially stable; however, after careful dosing of protamine, the patient exhibited hemodynamic lability. Epinephrine was increased to 10 mcg/min, and norepinephrine was started at 4 mcg/min and increased to 10 mcg/min. Vasopressin at 0.03 units/min was also added. During TEE examination of the LV via the deep trans-gastric view, a small, unremarkable amount of peri-hepatic fluid was seen. CPB was resumed and the decision was made to initiate veno-arterial extracorporeal membrane oxygenation (V-A ECMO) as a bridge to recovery. While the ECMO circuit was being primed, peak inspiratory pressures (PIP) were noted to be high at 28-30 cm H2O (20-21 cm H2O at baseline), and the surgeon informed us that the lungs were entering the mediastinum. In particular, the left lung was observed to be pushing the heart into the right chest, kinking of the SVG to the RCA, resulting in RV failure. Multiple causes were investigated and ruled out, including fluid in the left pleura, mainstem intubation, air trapping, reactive airway disease, and inadequate paralysis. Ventilation parameters were also changed in an attempt to limit lung impingement. Trans-gastric views of the liver on TEE now showed significant peri-hepatic fluid (Video 1, Figure 1), and the surgeon noted that the abdomen felt tight. An exploratory laparotomy was subsequently performed by general surgery. Upon initial opening of the peritoneum, ascitic fluid squirted up, at least 2-3 cm in height, through a small hole. Edematous bowel was found, and 8 L of clear ascitic fluid was released. At this point, the lungs ceased to impinge on the mediastinum, and CPB flows improved. The patient was diagnosed with ACS due to massive ascites. Her abdomen was left open, and a wound vacuum dressing was placed. LFTs were not checked. She was taken off CPB, and ECMO was initiated with adequate flows. After protamine re-administration, no clot formation was noted and all tissue surfaces continued to bleed briskly. A 500 cc of platelets and 3,900 cc of fresh frozen plasma (FFP) were given without improvement in coagulation status. We were unable to get TEG data or cryoprecipitate in a meaningful time frame. After discussing options with the surgeon and as a measure of last resort, 1,732.7666 units of prothrombin complex (PCC) were given. This dose was 25 mcg/kg, and the low dose indicated a reversal of supratherapeutic INR. The dosing of PCC is based on the factor IX component. Shortly after administration of PCC, ECMO flows decreased, and the surgeon observed bi-ventricular distension. The abdominal dressing was also noted to be bulging. The abdominal dressing was removed, revealing significant re-accumulation of ascites, compressing the IVC. In an attempt to improve venous drainage, the surgeon placed an additional venous ECMO cannula through the right femoral vein into the IVC. Despite these efforts, ECMO flows continued to decrease. Two episodes of ventricular fibrillation arrest with direct epicardial cardioversion then occurred. Clots were noted in the ECMO circuit and the left atrium on TEE (Video 2). The LV appeared very edematous, and the RV showed significant hypokinesis (Video 3). Given these findings, further resuscitative efforts were determined to be futile, and the time of death was called approximately 13 hours after induction of anesthesia.

**Video 1:** Peri-hepatic fluid on TEE

**Video 2:** Clot in heart seen on TEE

**Video 3:** Edematous and hypokinetic heart on TEE

Autopsy showed a markedly hypertrophic LV, a small LV chamber, and an overall small heart. There was no evidence of acute ischemic injury. Other than multi-organ edema, there were no significant pulmonary, hepatic, or renal pathologies.

## Discussion

Considering the risk factors and potential causes of ACS and the catastrophic IAH discussed here, it is important to ask, “What is it about cardiac surgery with CPB in this particular patient that led to an increased IAP and probable development of fatal ACS?”

Although we have no direct measurements of IAP for this case, it is clear that this patient had increased IAP and probable IAH. The abdomen was very distended and tense to palpation. Prior to surgery, the patient had a flat abdomen. When the peritoneum was opened, ascitic fluid squirted at least 2-3cm into the air through the small initial hole. This ascitic fluid was clearly under high pressure. In addition, the patient had no history of ascites/abdominal fluid prior to the surgery. Eight liters of ascitic fluid in an otherwise normal abdomen will, almost certainly, cause a dramatic rise in abdominal pressure. 

Poor venous drainage has been known to cause increased IVC pressure and ascites. In this patient, there was adequate venous drainage to maintain satisfactory CPB flows for the majority of the CPB run (until the development of ACS). Although we cannot rule out poor venous drainage as a cause of ACS in this case, it is unlikely to be the only cause. A three-stage cannula was used; the patient may have had adequate drainage from two of three cannula stages, resulting in adequate drainage for CPB, but may have had venous congestion distal to the third stage. As the ascites developed, they pushed the left lung into the mediastinum. Initially, this was mistakenly thought to be due to a lung ventilation issue, and it was not until a significant amount of ascites was seen on TEE, that the correct diagnosis was made and the abdominal pressure was relieved.

The patient also had a number of other known risk factors for ACS [1]. She was 69 years old. She had a long CPB exposure (cross-clamp time 164 min) at a temperature of 32℃. She required a large volume resuscitation (crystalloid and colloid) during and after CPB, resulting in a significant positive fluid balance and anemia. The long CPB time, administration of heparin and protamine twice, ascites, and possible hepatic failure, resulted in post-CPB bleeding and administration of significant blood and blood products. The patient developed a clear ascitic fluid, which can be seen with low protein content.

Escalating doses and types of inotropes and vasopressors are also associated with an increased risk of ACS [1]. At the time of the patient’s death, she was on large doses of epinephrine, norepinephrine, and vasopressin. As the duration of CPB increased and with each failed attempt to separate from CPB, more inotropes were added, which increased the risk for IAH and ACS.

An increased inflammatory response to CPB is also a risk factor for ACS with CPB. As it is not standard of care and we did not consider excessive inflammation as a cause of the ascites, we did not draw any inflammatory markers; therefore, we cannot determine if this is a cause of IAH and ACS in this case. Additionally, as inflammation was not high on our differential at the time, we did not administer steroids, which may have been helpful in this case.

An increased baseline IAP is a risk factor for the development of IAH and ACH. As we had a standard temperature monitoring device (a urinary bladder catheter), we were unable to easily monitor IAP. Monitoring IAP from the indwelling catheter would have involved a complex process and set up for transducing pressures from the catheter aspiration point and clamping the drainage line while the patient was fully prepped and draped for open-heart surgery. An alternative would have been placing a catheter in the abdomen while it was still closed. Once we determined that the abdomen was distended, the primary goal was to open the open abdomen, release the pressure and perhaps determine the cause. At this point, we did not have access to bladder catheters that measure IAP. For these reasons, we have no IAP readings from this case. Once massive ascites developed, the diaphragm elevated, and the lung pushed into the mediastinum, it is clear that IAP was elevated. This was confirmed when the abdomen was open and ascitic fluid squirted out of a small peritoneal incision under high pressure.

Another possible cause of IAH and ACS, in this case, may have been ischemia. Lower coronary perfusion pressures resulted in myocardial dysfunction and heart failure. Cardiac catheterization prior to surgery showed significant RCA disease and no significant left-sided disease. This resulted in an initial bypass of only the RCA. Given the fact that disease was directly palpated on the LAD and LCxA, the degree of CAD in the left coronary distribution was underestimated at the time of catheterization, and this discrepancy was not communicated to the anesthesia team. Had this disease been clearly communicated, ischemia that was thought to be due to air would have been more likely to be correctly diagnosed as due to CAD. The 45 minutes spent trying to eliminate “air” was a significant time for ischemia to result in cardiac edema, decreased myocardial perfusion, global heart failure, and resulting ascites. Additionally, the staged grafting of the RCA and then the LAD resulted in a longer CPB time, which is a risk factor for ACS (as discussed above). We have no lactate levels with our arterial blood gasses, so we have no additional information as to the degree of acidosis potentially caused by tissue (especially myocardial) hypoperfusion.

## Conclusions

Our patient had a number of the risk factors for the development of IAH and ACS described by Dalfino et al.: she was 69 years old, her baseline CVP was 12, she underwent a long duration of hypothermic CPB, had a significantly positive fluid balance, was exposed to vasoactive drugs, probably had a significant period with cardiac ischemia and may have had poor venous drainage during CPB. Unfortunately, her baseline IAP was not known and inflammatory markers were not drawn during the case. Return to CPB resulted in this older patient being further exposed to these risk factors. The patient then developed myocardial edema, heart failure, and coagulopathy. ECMO, massive transfusion therapy, and PCC administration were all attempted as resuscitation and salvage methods, without success.

This case demonstrates how prompt recognition of this rare event and quick initiation of support is necessary to adequately treat this potentially deadly complication. Since this event, our institution has converted to the use of bladder catheters that have the ability to monitor IAP. These catheters measure IAP via a pressure sensor in the catheter and as a second balloon that is transiently able to occlude the urinary catheter to allow for pressure measurements in the bladder. In an empty bladder, bladder pressures are essentially equivalent to IAP. However, even with prompt recognition and treatment, this condition is often fatal. Some factors, such as the degree of the inflammatory response to CPB, are almost impossible to predict and promptly measure at this time. Limiting fluid administration on CPB may be feasible with the use of vasopressors, but vasopressors themselves are a risk factor for IAH in this patient population. Other factors such as age, weight, and baseline cardiac function are difficult, if not impossible, to alter pre-operatively. Successful treatment may rely on knowledge of the syndrome and its risk factors, a high index of suspicion, prompt treatment, and a fair amount of luck.
